# Genome-wide analysis reveals the MORC3-mediated repression of PD-L1 expression in head and neck cancer

**DOI:** 10.3389/fcell.2024.1410130

**Published:** 2024-09-12

**Authors:** Wenxuan Fu, Xiaomeng Chang, Kun Ye, Zige Zheng, Qianyi Lai, Minyang Ge, Yan Shi

**Affiliations:** ^1^ School of Stomatology, Nanchang University, Nanchang, China; ^2^ Jiangxi Province Key Laboratory of Oral Biomedicine, Nanchang, China; ^3^ Jiangxi Province Clinical Research Center for Oral Diseases, Nanchang, China

**Keywords:** MORC3, PD-L1, head and neck cancer, cell proliferation, LINC00880

## Abstract

**Introduction:**

Programmed death-ligand 1 (PD-L1) plays essential roles in the negative regulation of anti-tumor immunity. However, the regulatory mechanisms of PD-L1 expression need further exploration. MORC family CW-type zinc finger 3 (MORC3) is a transcriptional factor that regulates innate immune responses, but the expression and roles of MORC3 in cancers remain largely unknown. The present study explored the expression of MORC3 in cancers at both transcriptional and translational levels.

**Methods:**

The target genes and pathways were analyzed using RNA interference (RNAi), RNA sequencing (RNA-seq), and quantitative real-time polymerase chain reaction (qRT-PCR) technology in head and neck cancer cells. The expression of MORC3 and its target genes were also analyzed in single cancer cells.

**Results:**

MORC3 was significantly downregulated in multiple cancers, including head and neck cancer, and low expression of MORC3 was associated with poor overall survival. MORC3 knockdown significantly increased the expression of many immune-related genes, including interferon (IFN)-associated genes [MX dynamin like GTPase 2 (MX2), interferon induced protein with tetratricopeptide repeats 1 (IFIT1), interferon induced protein with tetratricopeptide repeats 2 (IFIT2), interferon regulatory factor 7 (IRF7), interferon regulatory factor 9 (IRF9), interferon induced protein 44 like (IFI44L), interferon induced transmembrane protein 1 (IFITM1), interferon induced transmembrane protein 3 (IFITM3), interferon induced protein 44 (IFI44), and interferon induced with helicase C domain 1 (IFIH1)]. MORC3 knockdown significantly upregulated PD-L1 and signal transducer and activator of transcription 1 (STAT1) expression. Moreover, the LINC00880 immune-related long non-coding RNA (lnc-RNA) was upregulated by MORC3 knockdown. Silencing LINC00880 attenuated PD-L1 expression. MORC3 knockdown also increased the expression of cellular proliferation-related genes and promoted cancer cell proliferation.

**Conclusion:**

The present study demonstrated that MORC3 regulates IFN-associated pathways and is a novel repressor of PD-L1 expression and cancer cell proliferation.

## 1 Introduction

MORC family CW-type zinc finger 3 (MORC3; also known as NXP2 and ZCWCC3) belongs to the microrchidia (MORC) family, which also includes MORC1, MORC2, and MORC4. The MORC family contains several conserved domains, including a GHKL-ATPase domain at the N-terminus, a CW-type zinc finger domain, and a coiled-coil domain at the C-terminus. The MORC3 protein has two distinct domains, namely, a nuclear matrix-binding domain and an RNA-binding domain (Hong et al., 2017). In addition, the CW structural domain of the MORC3 recognizes histone H3 trimethylated at Lys-4 (H3K4me3) at the promoter of target genes and represses transcription (Li et al., 2016; Desai et al., 2021). Knockout of Morc3 in mice is lethal (Takahashi et al., 2007). MORC3 is widely expressed in multiple tissues and cells (Wang et al., 2021). MORC3 is a multifunction protein and is involved in the inhibition of viral gene transcription (Ma et al., 2022), epigenetic regulation of gene expression (Li et al., 2016), and induction of cellular senescence (Takahashi et al., 2007). Moreover, MORC3 functions as a negative regulator of interferon beta 1 (IFNB1) in monocytes independent of canonical immune pathways (Gaidt et al., 2021). MORC3 is also associated with autoimmune diseases (Gunawardena et al., 2009) and Down syndrome (Andrews et al., 2016). Anti-MORC3 antibodies have been found in Down syndrome patients with cancer, and MORC3 is genetically changed in multiple cancers (Andrews et al., 2016). However, the expression, roles, and targets of MORC3 in cancers, including head and neck cancer, remain largely unknown.

Head and neck cancer is a common malignancy arising from the mucosal epithelia of the head and neck region. Programmed death-ligand 1 (PD-L1; also called CD274 and B7-H1) is an immune checkpoint molecule, and it is highly expressed in head and neck cancer (Steen et al., 2023). Increased expression of PD-L1 in oral squamous cell carcinoma (OSCC) is associated with metastasis and poor clinical outcome (Maruse et al., 2018). PD-L1 plays essential roles in the negative regulation of immune responses by binding to the programmed cell death protein 1 (PD-1) protein on the surface of T cells. Immunotherapy targeting the anti-PD-1/PD-L1 axis is used to treat recurrent or metastatic head and neck squamous cell carcinoma (HNSCC) (Harrington et al., 2023). Although anti-PD-1/PD-L1 immunotherapy is promising in the treatment of multiple cancers, most patients still experience primary or acquired resistance. Therefore, it is important to study the regulatory mechanisms of PD-1/PD-L1. The expression of PD-L1 can be regulated at the genetic, epigenetic, transcriptional, and translation levels (Lin et al., 2023). However, the regulatory mechanisms of PD-L1 expression need further exploration to facilitate the discovery of novel anti-PD-L1 treatments.

The present study explored the expression levels of MORC3 mRNA in The Cancer Genome Atlas (TCGA) cancers and MORC3 protein in head and neck cancer. The present study also investigated MORC3 target genes and pathways by knocking down MORC3 expression, performing RNA-sequencing (RNA-seq) analysis of genome-wide transcriptome, and verifying expression using quantitative real-time polymerase chain reaction (qRT-PCR) in the CAL 27 head and neck cancer cell line. The expression levels of MORC3 and its target genes were further confirmed by single-cell RNA-seq analysis of head and neck cancer cells. The present data revealed new targets of MORC3 and elucidated the ability of MORC3 to suppress PD-L1 expression in head and neck cancer cells. The present findings may provide novel anti-PD-L1 immunotherapy strategies in cancers.

## 2 Materials and methods

### 2.1 Cells and plasmids

CAL 27 cells were cultured in Dulbecco’s modified Eagle medium (DMEM, Cytiva, Shanghai, China) supplemented with 10% fetal bovine serum (FBS, Cellmax, Lanzhou, China). The human MORC3 expression plasmid was obtained from VectorBuilder (Guangzhou, China).

### 2.2 RNAi

The anti-MORC3 (human) and non-specific siRNAs were synthesized by Sangon (Shanghai, China). The anti-MORC3 siRNA sequence was 5′- GUG​AGG​UUG​AAU​UGC​UGG​AAA-3′, and the anti-LINC00880 siRNA sequence was 5′- AUC​UUU​UUA​GCA​UCA​UAG​ACA​CU-3’. The siRNAs were transfected into CAL 27 cells using Lipofectamine 3000 (Invitrogen, Waltham, MA, United States) according to the manufacturer’s instructions.

### 2.3 Immunohistochemistry

The tissue microarray purchased from Alenabio company (Xi’an, China) included 48 OSCC tissues and 10 normal oral mucosal tissues (Supplementary Table S1). The tissue microarray was deparaffinized by xylene and then rehydrated using a series of graded ethanol. The microarray was then incubated with Target Retrieval Solution (Dako, Glostrup, Denmark), and endogenous peroxidase was inactivated by incubation with Peroxidase-Blocking Reagent (Dako). The microarray was then incubated with mouse monoclonal anti-MORC3 antibody (Santa Cruz Biotechnology, Dallas, TX, United States) overnight at 4°C, followed by incubation with the FLEX/HRP secondary antibody (Dako) for 30 min. MORC3 expression was visualized after incubation with 3,3′-diaminobenzidine (DAB, Dako) and scanning with a Panoramic MIDI (3D HISTECH, Hungary).

### 2.4 Bioinformatics analysis

The expression of MORC3 in TCGA cancers was analyzed using the Gene Expression Profiling Interactive Analysis 2 (GEPIA2) online database, with a |log2 (fold-change)| cutoff of 0.2 and *p*-value of 0.05. The clinical outcomes of head and neck cancer male patients were downloaded from the Human Protein Atlas (HPA) database.

The expression levels of MORC3 and interferon (IFN)-associated genes in single cells of head and neck cancer reported in a previous study (Puram et al., 2017) were downloaded using the CancerSEA database. Cells expressing MORC3 and/or IFN-associated genes were used for correlation analyses using GraphPad Prism 8.

### 2.5 RNA-seq analysis

Total RNA was purified from CAL 27 cells treated with anti-MORC3 siRNA or non-specific siRNA. RNA-seq and corresponding bioinformatics analyses were performed by Wuhan Ruixing Biotechnology Co. Ltd. (Wuhan, China). In brief, 1 µg of total RNA was used for RNA-seq library preparation, followed by high-throughput sequencing with the Illumina Novaseq 6,000 system. The alignment was performed with HISAT2 software (version 2.1.0). The expression level of each gene was calculated by fragments per kilobase of transcript per million mapped reads (FPKM), and the differentially expressed genes (DEGs) were identified by DESeq2 software (version 1.34.0) based on the criteria of fold change ≥3/2 or ≤2/3 and *p*-value <0.05. The function and pathway analyses of the DEGs were performed using Reactome and Kyoto Encyclopedia of Genes and Genomes (KEGG) enrichment programs. Enriched pathways were further evaluated using gene set enrichment analysis (GSEA) to verify the corresponding pathways based on the expression of all genes.

### 2.6 Reverse transcription-PCR (RT-PCR) and qRT-PCR

Total cellular RNA was reverse transcribed using Maxima H Minus cDNA Synthesis Master Mix (Thermo Fisher, United States). The cDNA was amplified using Green Taq Mix (Vazyme Biotech, Nanjing, China) for PCR using the following primers: 5′- ACA​AAT​CAA​CAG​ACG​GCA​ACA​G-3′ and 5′-CAT​AGC​CAG​CAG​TTG​TCC​TAC​G-3′ for MORC3; and 5′- GAA​GGT​GAA​GGT​CGG​AGT​C-3′ and 5′-GAA​GAT​GGT​GAT​GGG​ATT​TC-3′ for GAPDH. The cDNA was used for qRT-PCR using ChamQ Universal SYBR qPCR Master Mix (Vazyme Biotech, Nanjing, China) with specific primers (Supplementary Table S2). The relative expression level of each gene was computed by the 2^−ΔΔCT^ method, in which GAPDH was used as an internal control for normalization.

### 2.7 Western blot analysis

Cells were lysed in 2 x sodium dodecyl sulfate (SDS) sampling buffer. Total cellular protein samples were denatured for 3 min at 95°C and separated using 10% SDS-polyacrylamide gel electrophoresis (PAGE), and proteins were then transferred to nitrocellulose membranes. The membranes were blocked with 5% skimmed milk and incubated overnight at 4°C with following antibodies: mouse monoclonal anti-MORC3 antibody (Santa Cruz Biotechnology, United States), rabbit monoclonal anti- interferon induced protein with tetratricopeptide repeats 2 (IFIT2) antibody (abcam, United States), rabbit monoclonal anti-interferon induced transmembrane protein 3 (IFITM3) antibody (Cell Signaling Technology, United States), rabbit monoclonal anti-interferon induced protein with tetratricopeptide repeats 1 (IFIT1) antibody (Cell Signaling Technology, United States), rabbit monoclonal anti-cyclin D1 (CCND1) (ABclonal, China), rabbit monoclonal anti-cyclinD2 (CCND2) (ABclonal, China), rabbit polyclonal anti-JUN (ABclonal, China), rabbit polyclonal anti-interferon regulatory factor 7 (IRF7) (Proteintech, China), rabbit polyclonal anti-PD-L1 antibody (Proteintech, China), rabbit polyclonal anti-DExD/H-Box Helicase 60 (DDX60) antibody (Proteintech, China), and mouse anti-β-actin (Abmart, China). The membranes were then incubated with horseradish peroxidase (HRP)-conjugated anti-mouse or anti-rabbit IgG antibody. Bound antibody was visualized using an enhanced chemiluminescence (ECL) kit (Merck Millipore) and X-ray films. β-actin was used as an internal loading control.

### 2.8 Cell proliferation assay

CAL 27 cells were seeded into a 12 well-plate (1 × 10^6^ cells per well). After 2 h, the cells were transfected with anti-MORC3 or non-specific (NS) siRNA using Lipofectamine 3000 (Invitrogen). At Days 2 and 4 after transfection, cells were counted using the trypan blue exclusion method.

### 2.9 Flow cytometry

CAL 27 cells were treated with anti-MORC3 (human) siRNA and non-specific siRNA for 48 h. The expression of PD-L1 on the surface of CAL 27 cells was analyzed by flow cytometry using an allophycocyanin (APC)-labeled anti-human PD-L1 antibody (eBioscience, United States) and a CytoFlex (Beckman, United States).

### 2.10 Statistical analysis

Two-group statistical comparisons were performed by a Student’s t-test using GraphPad Prism 8. *p* < 0.05 was considered statistically significant.

## 3 Results

### 3.1 MORC3 expression levels are low in cancer

**FIGURE 1:**
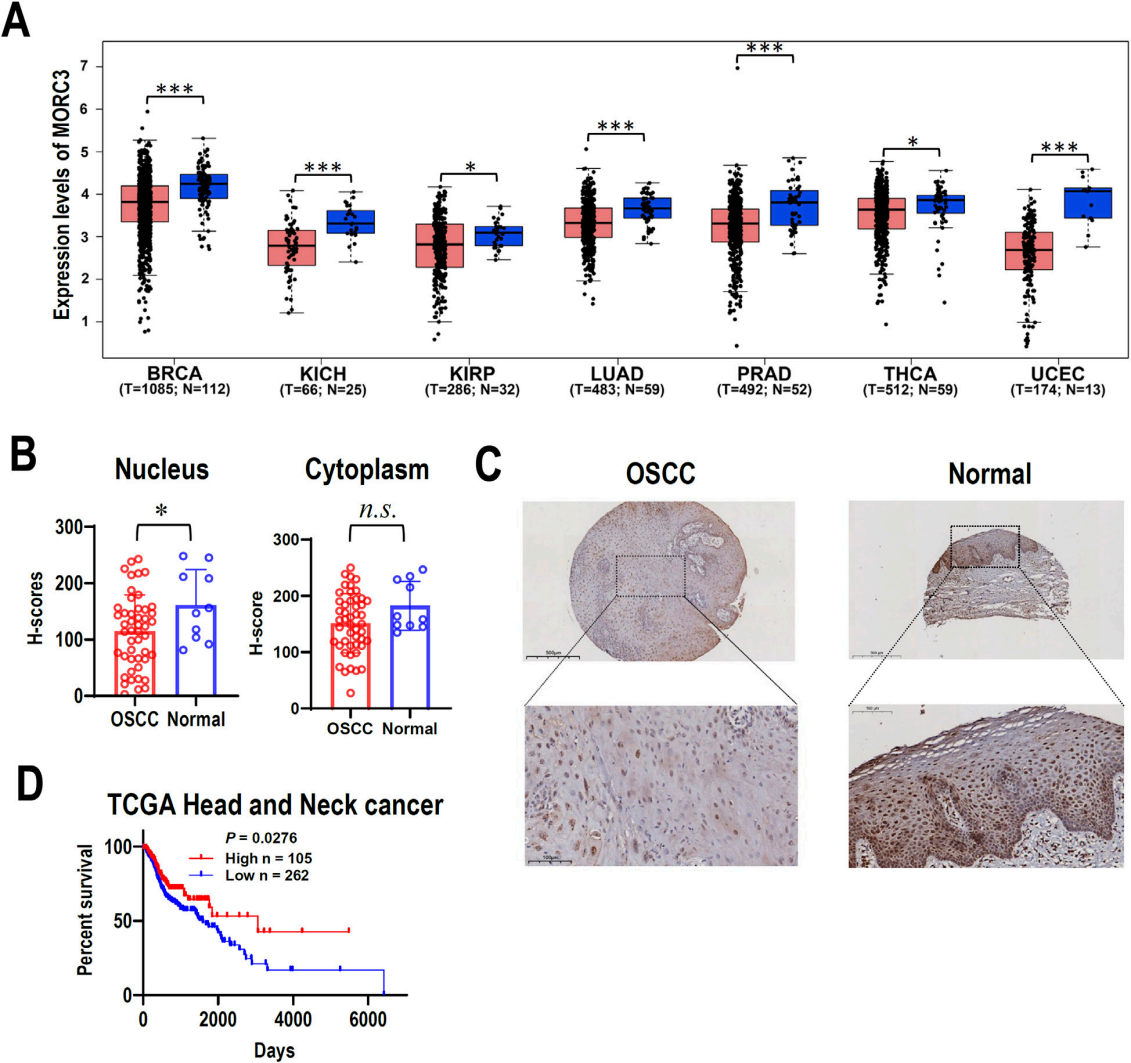
The expression of MORC3 in cancers. **(A)** The transcriptional expression levels of MORC3 in multiple cancers of TCGA database were analyzed by using GEPIA2 online service. The cancer types include breast (BRCA), kidney (KICH and KIRP), lung (LUAD), prostate (PRAD), thyroid (THCA), and uterine (UCEC) cancers. **(B, C)** The cytoplasmic or nucleic protein expression levels of MORC3 in a tissue microarray containing oral squamous cell carcinoma (OSCC, n = 48) and normal oral mucosal tissues (n = 10). Panel C showed the representative immunostaining of MORC3 in OSCC and normal tissue. **(D)** Kaplan Meier survival curve of TCGA HNSCC patient (male) with high-expression (n = 105) or low-expression (n = 262) of MORC3. *, *p* < 0.05; ***, *p* < 0.001, n.s., no significance.

MORC3 mRNA expression was investigated in TCGA cancers. The expression of MORC3 in seven cancer tissues, including breast, kidney, lung, prostate, thyroid, or uterine cancers, was significantly lower compared to the expression in corresponding normal control tissues (Figure 1A). The expression of MORC3 protein was analyzed in a tissue microarray containing 48 OSCC tissues and 10 normal oral mucosal tissues by immunohistochemistry. The expression of MORC3 was significantly lower in the nucleus where MORC3 is normally expressed, but not the cytoplasm, of OSCC cancer cells compared to epithelial cells in normal mucosal tissues (Figures 1B, C). In addition, male patients in TCGA head and neck squamous cell carcinoma (HNSCC) with high MORC3 expression showed significantly longer survival than those with low MORC3 expression (Figure 1D). These results suggested that MORC3 has low expression in cancer and may be associated with favorable prognosis.

### 3.2 MORC3 mediates target gene expression

**FIGURE 2:**
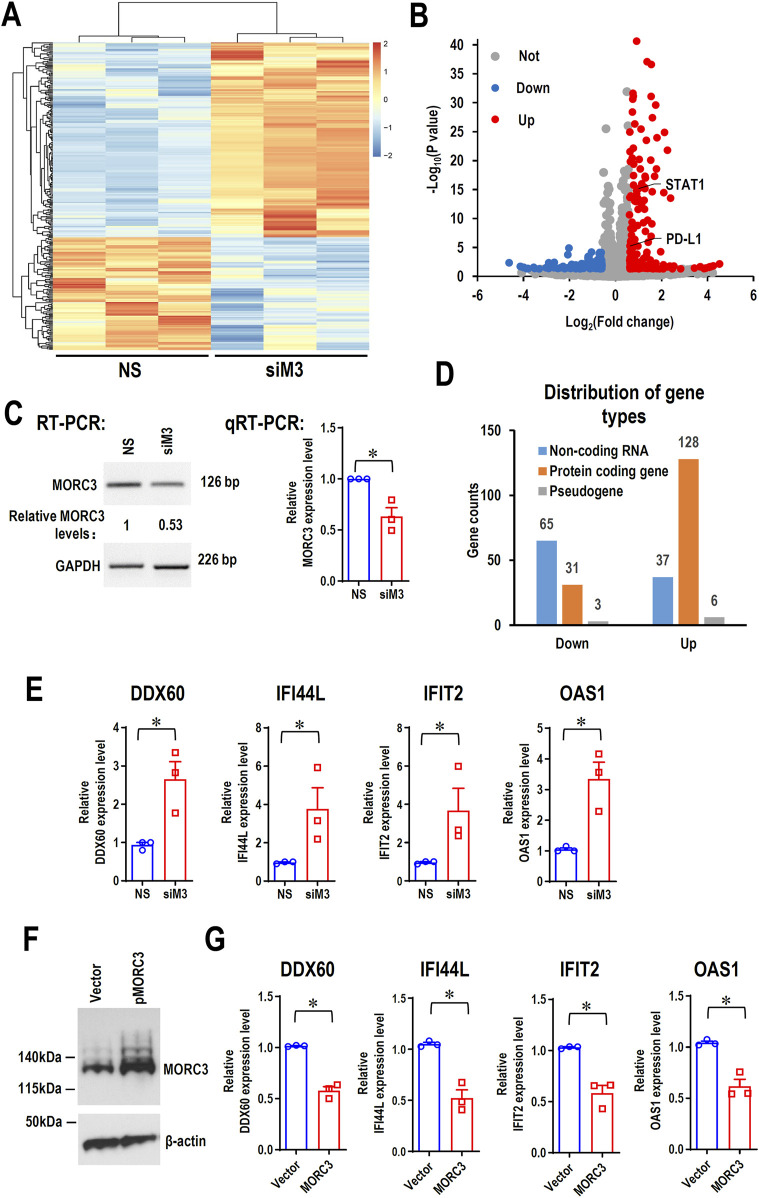
The RNA-seq analysis of the differentially expressed genes (DEGs) between anti-MORC3 siRNA (siM3) and non-specific siRNA in CAL 27 cells. **(A)** Heat map of the differentially expressed genes. **(B)** Volcano plot of the differentially expressed genes. **(C)** Confirmation of MORC3 knockdown by RT-PCR or qRT-PCR. GAPDH served as a loading control. **(D)** Distribution of gene types of the differentially expressed genes. **(E)** The expression levels of DDX60, IFI44L, IFIT2, and OAS1 in cells with or without MORC3 knockdown were verified by qRT-PCR. **(F)** Overexpression of MORC3 was confirmed by Western blot in CAL 27 cells. **(G)** The expression levels of DDX60, IFI44L, IFIT2, and OAS1 in cells with or without MORC3 overexpression were analyzed by qRT-PCR. *, *p* < 0.05.

To elucidate the potential roles of MORC3 at a genome-wide level, MORC3 was knocked down in the CAL 27 OSCC cell line, and the molecular targets of MORC3 were explored by RNA-seq. In total, 270 significantly differentially expressed genes (DEGs) (171 upregulated and 99 downregulated) were identified after MORC3 knockdown (Figures 2A–C). Among these DEGs, the expression levels of most protein-coding genes increased, whereas the expression levels of most non-coding RNA genes decreased (Figure 2D). Four target genes, namely, DDX60, interferon induced protein 44 like (IFI44L), interferon induced protein with tetratricopeptide repeats 2 (IFIT2), and 2′-5′-oligoadenylate synthetase 1 (OAS1), were randomly selected for verification. In line with the RNA-seq results, qRT-PCR analysis demonstrated that these genes were significantly upregulated after MORC3 knockdown (Figure 2E). In contrast, overexpression of MORC3 significantly decreased the expression levels of these genes (Figures 2F–G; Supplementary Figure S1). Furthermore, the protein expression levels of DDX60 and IFIT2 were also upregulated after MORC3 knockdown but downregulated after MORC3 overexpression (Supplementary Figure S2). These results demonstrated that MORC3 regulates the expression of target genes, including both coding and noncoding genes.

### 3.3 MORC3 is significantly involved in immune-associated pathways

**FIGURE 3:**
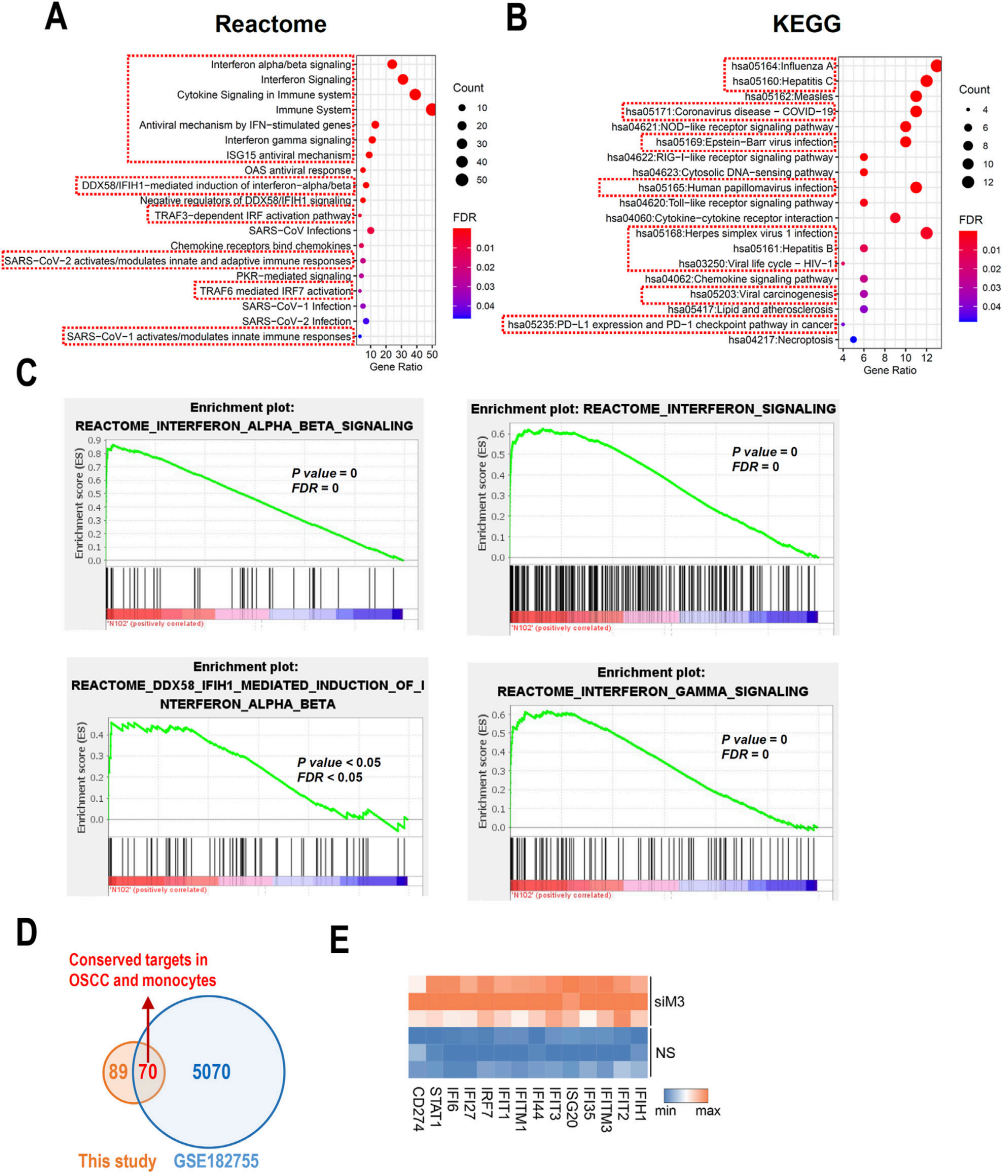
Pathway analysis based on DEGs. **(A)** Bubble plots shows the functional enrichment Reactome analysis of DEGs. The pathways related to immune regulation were highlighted by red boxes. **(B)** Bubble plots shows the functional enrichment KEGG analysis of DEGs. The pathways related to virus infection were highlighted by red boxes. **(C)** The GSEA analysis of the IFN- associated signaling pathways. **(D)** The conserved gene targets of MORC3 between this study of HNSC and a study performed in monocytes from GSE182755. **(E)** Heat map of significantly changed interferon-associated genes.

To further understand the potential roles of MORC3, pathway analyses of significant DEGs were performed. Reactome pathway analysis showed that MORC3 may be involved in multiple immune regulatory pathways, especially many IFN-associated pathways, including IFNα/β/γ signaling and anti-viral mechanisms regulated by IFN-stimulated genes and ISG15 (Figure 3A). KEGG pathway enrichment analysis showed that MORC3 may regulate multiple virus-related pathways, including influenza A, hepatitis B, hepatitis C, COVID-19, Epstein-Barr virus, human papillomavirus, herpes simplex virus 1, and human immunodeficiency virus 1 (HIV-1). Importantly, viral carcinogenesis, PD-L1 expression, and the PD-1 checkpoint pathway in cancer were also enriched (Figure 3B). KEGG analysis also identified the involvement of MORC3 in the retinoic acid-inducible gene I (RIG-I)-like receptor signaling pathway, Toll-like receptor signaling pathway, and chemokine signaling pathway. GSEA confirmed that these immune- and IFN-associated pathways were significantly activated after MORC3 knockdown (Figure 3C; Supplementary Figure S3). MORC3 may also regulate cytosolic DNA-sensing pathway by increasing the expression of Z-form nucleic acid-binding protein 1 (ZBP1), a DNA and RNA sensor, as well as DEAD box protein 58 (DDX58, also called RIG-1). The target genes identified in the present study were compared with the target genes identified in the GSE182755 database, which contains data from MORC3-deficient monocytes. In total, 70 targets were shared by both OSCC cells and monocytes, indicating that these genes may be conserved across cell types (Figure 3D). Among these genes, interferon stimulated exonuclease gene 20 (ISG20), PD-L1 (CD274), signal transducer and activator of transcription 1 (STAT1), interferon induced with helicase C domain 1 (IFIH1), IRF7, interferon induced protein with tetratricopeptide repeats 2 (IFIT2), interferon induced protein 35 (IFI35), IFIT1, interferon induced protein 44 (IFI44), interferon induced protein with tetratricopeptide repeats 3 (IFIT3), interferon induced transmembrane protein 1 (IFITM1), interferon alpha inducible protein 27 (IFI27), interferon induced transmembrane protein 3 (IFITM3), and interferon alpha inducible protein 6 (IFI6) were upregulated in MORC3-deficient cells and were closely related to IFN-associated pathways (Figure 3E).

### 3.4 MORC3 is significantly involved in IFN-associated pathways

**FIGURE 4:**
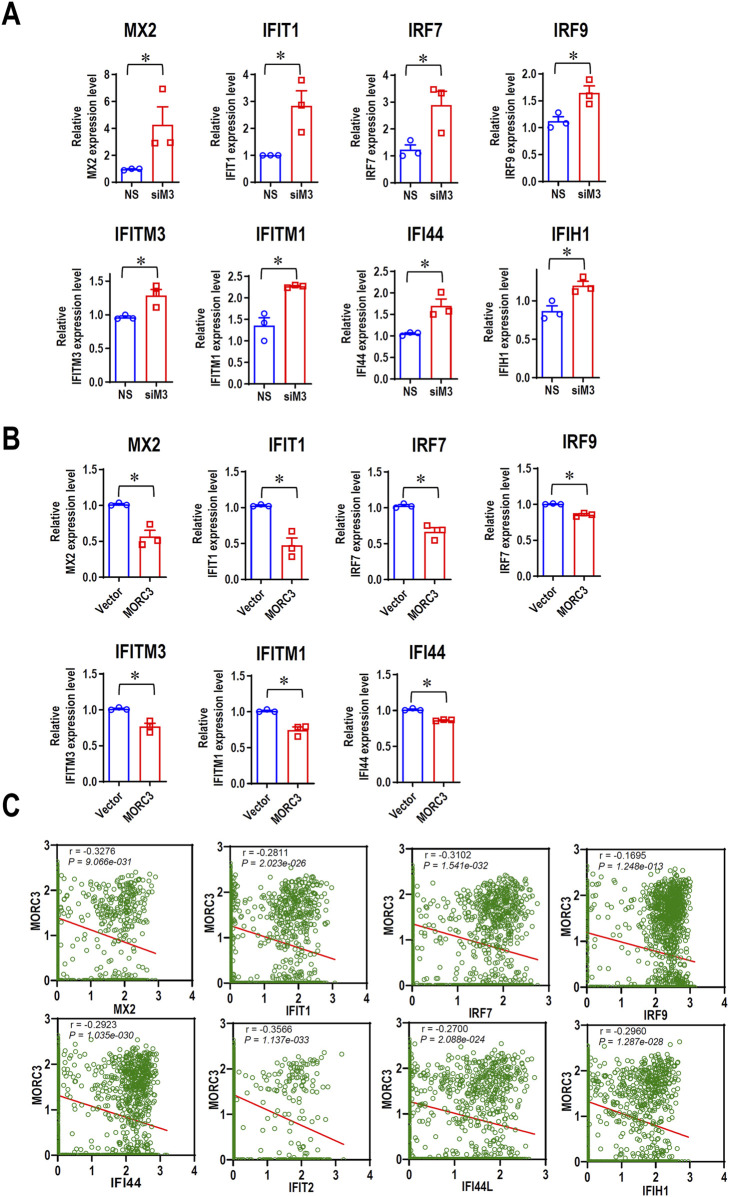
MORC3 represses the expression of many IFN-associated target genes. **(A)** The expression levels of IFN-associated target genes MX2, IFIT1, IRF7, IRF9, IFITM3, IFITM1, IFI44, and IFIH1 in CAL 27 cells with or without MORC3 knockdown were analyzed by qRT-PCR. **(B)** The expression levels of IFN-associated target genes MX2, IFIT1, IRF7, IRF9, IFITM3, IFITM1, and IFI44 in CAL 27 cells with or without MORC3 overexpression were analyzed by qRT-PCR. **(C)** The correlation between the expression levels of MORC3 and IFN-associated target genes in single cells of head and neck cancer were evaluated by reanalyzing the expression data of GSE10332 in the database of CancerSEA. *, *p* < 0.05.

To further elucidate the roles of MORC3 in the expression of IFN-associated genes in HNSC cells, qRT-PCR was used to evaluate the expression of additional target genes associated with IFN in CAL 27 cells. In MORC3-deficient cells, the expression levels of MX dynamin like GTPase 2 (MX2), IFIT1, IRF7, interferon regulatory factor 9 (IRF9), IFITM3, IFITM1, IFI44, and IFIH1 were significantly upregulated compared to control cells (Figure 4A). In MORC3-overexpressing cells, the expression levels of MX2, IFIT1, IRF7, IRF9, IFITM3, IFITM1, and IFI44 were significantly downregulated compared to control cells (Figure 4B). Moreover, the protein expression levels of IRF7, IFIT1, and IFITM3 were upregulated after MORC3 knockdown, but downregulated after MORC3 overexpression (Supplementary Figure S4). The expression correlation between MORC3 and these genes were analyzed at the single-cell level in head and neck squamous cell carcinoma using the GSE103322 database. The expression level of MORC3 was significantly negatively associated with MX2, IFIT1, IRF7, IRF9, IFI44, IFIT2, IFI44L, and IFIH1 in HNSCC cells (Figure 4C). These results indicated that MORC3 represses IFN-associated pathways in OSCC, which may contribute to tumor suppression because weak and persistent IFN responses support tumorigenesis.

### 3.5 MORC3 decreases STAT1 and PD-L1 expression

**FIGURE 5:**
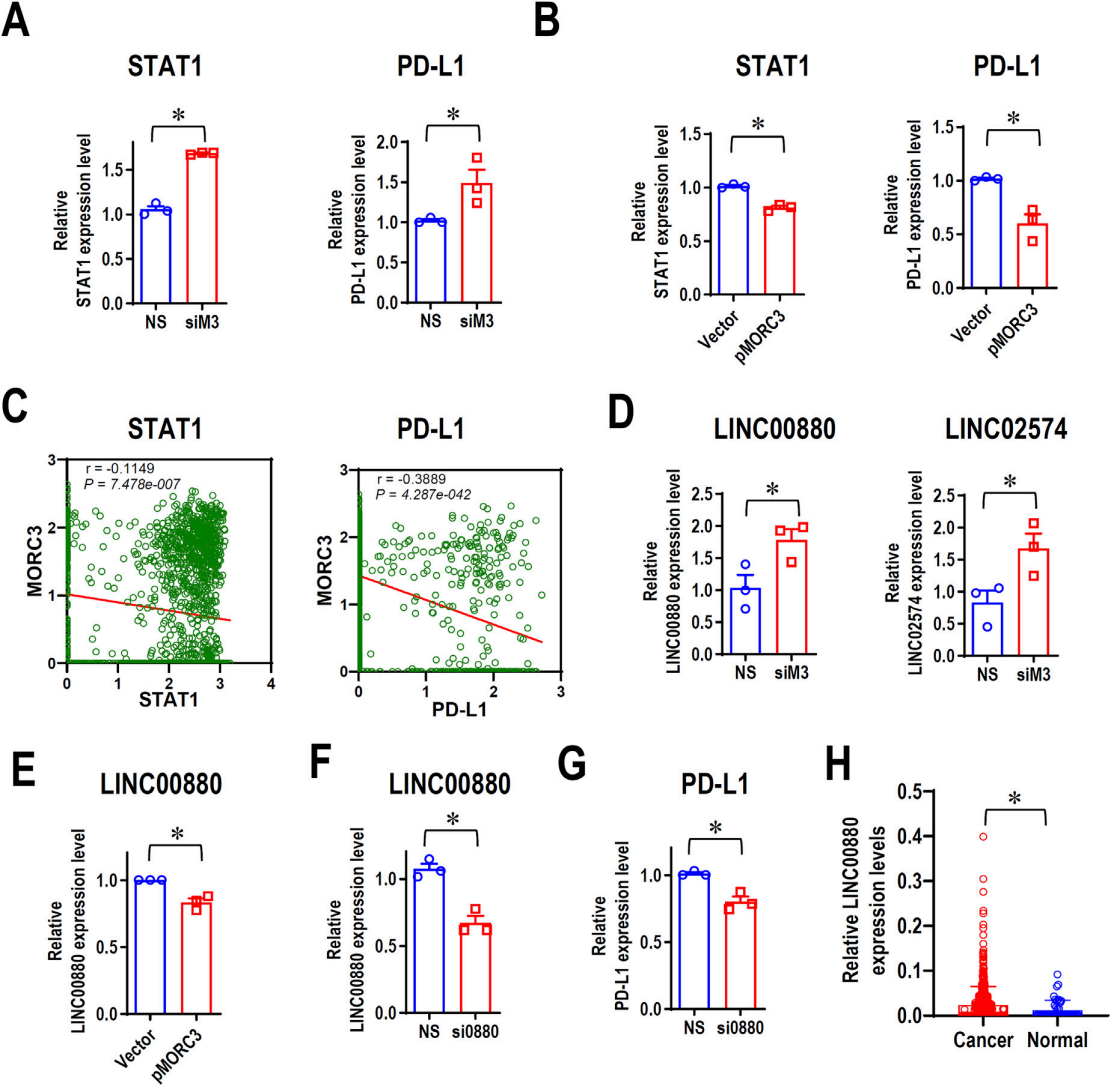
MORC3 represses PD-L1 expression. **(A)** The expression levels of STAT1 and PD-L1 in CAL 27 cells with or without MORC3 knockdown were analyzed by qRT-PCR. **(B)** The expression levels of STAT1 and PD-L1 in CAL 27 cells with or without MORC3 overexpression were analyzed by qRT-PCR. *, *p* < 0.05. **(C)** The correlation between the expression levels of MORC3 and STAT1 and PD-L1 in single cells of OSCC were analyzed by reanalyzing the expression data of GSE10332 in the database of CancerSEA. **(D)** The expression levels of lncRNA LINC00880 and LINC02574 in CAL 27 cells with or without MORC3 knockdown were analyzed by qRT-PCR. **(E)** qRT-PCR analysis of LINC00880 expression in CAL 27 cells with or without MORC3 knockdown. **(F)** qRT-PCR analysis of the efficiency of LINC00880 knockdown by siRNA. Si0880 is a siRNA against LINC00880. **(G)** The expression level of PD-L1 in CAL 27 cells with or without LINC00880 knockdown was analyzed by qRT-PCR. *, *p* < 0.05. **(H)** The expression levels of LINC00880 in TCGA HNSCC were analyzed in OncoDB database.

The PD-L1 immune checkpoint is a major negative regulator of immune response and is overexpressed in cancer, and the IFNγ-STAT1 pathway mediates the expression of PD-L1 (Lv et al., 2021). RNA-seq demonstrated that MORC3 knockdown significantly increased STAT1 and PD-L1 expression, which was confirmed by qRT-PCR analysis (Figure 5A). Flow cytometry analysis confirmed that MORC3 deficiency significantly increased the levels of PD-L1 protein on the surface of CAL 27 cells (Supplementary Figure S5). In contrast, MORC3 overexpression significantly decreased STAT1 and PD-L1 expression (Figure 5B). In addition, the expression level of MORC3 was significantly negatively associated with STAT1 and PD-L1 at the single-cell level in HNSCC (Figure 5C). These results demonstrated that MORC3 represses the expression of PD-L1 and its upstream activator.

### 3.6 MORC3 represses the expression of the LINC00880 long non-coding RNA (lnc-RNA) and PD-L1

MORC3 targets several lncRNAs, including LINC00880, which is overexpressed in multiple cancers (Feng et al., 2023), and LINC02574, which enhances the innate immune response (Zhang et al., 2023). RNA-seq analysis demonstrated that the expression levels of both lncRNAs increased after MORC3 knockdown, and qRT-PCR analysis confirmed these results (Figure 5D). In contrast, MORC3 overexpression significantly decreased LINC00880 expression (Figure 5E). Silencing LINC00880 by siRNA (Figure 5F) significantly decreased the expression of PD-L1 at both the mRNA (Figure 5G) and protein levels (Supplementary Figure S6). Further, LINC00880 was overexpressed in head and neck cancer (Figure 5H). These findings indicated that MORC3 represses PD-L1 expression via multiple pathways, including through the downregulation of LINC00880.

### 3.7 MORC3 knockdown promotes cancer cell proliferation

**FIGURE 6:**
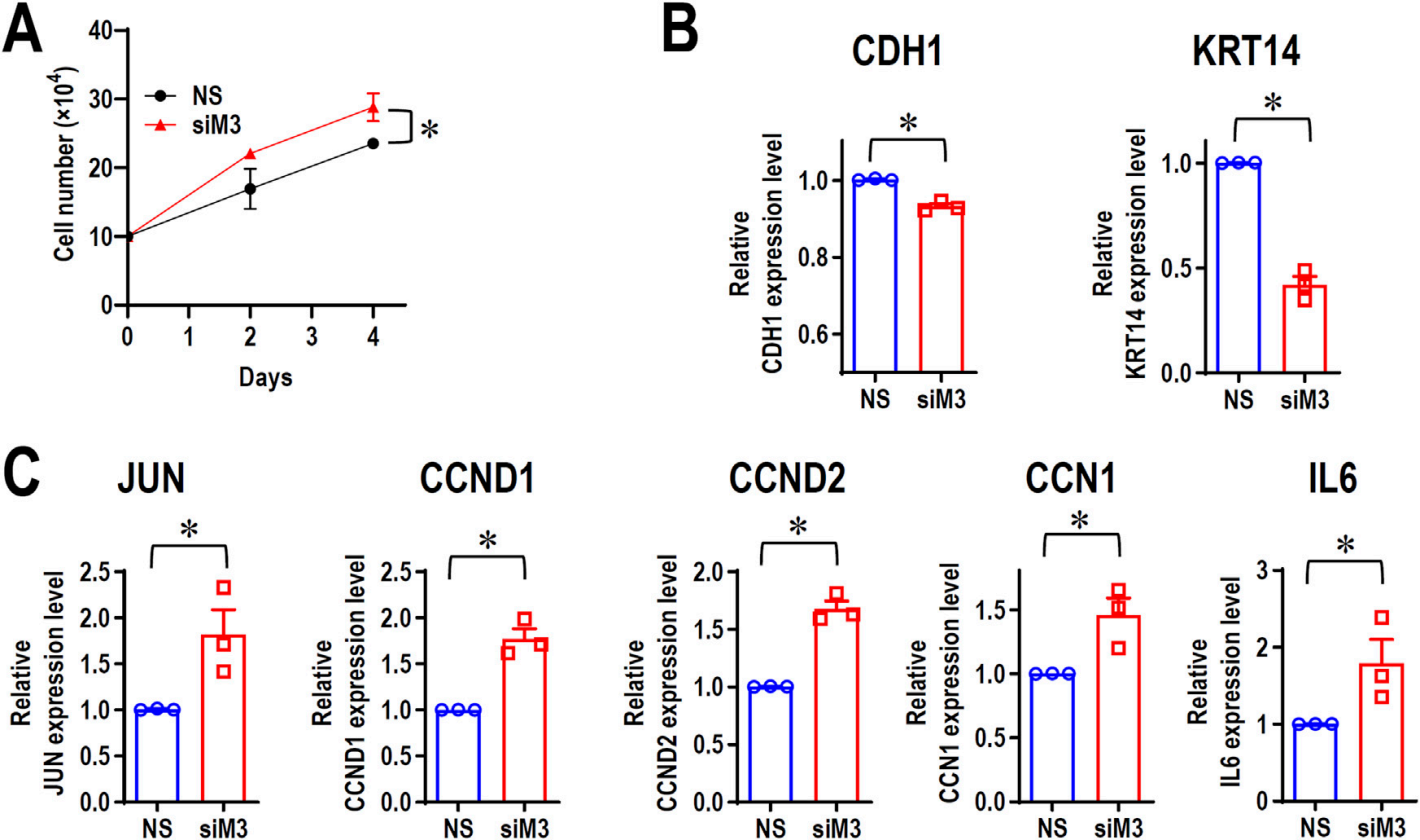
MORC3 plays negative roles in cancer cell proliferation. **(A)** CAL 27 cells were transfected with anti-MORC3 or non-specific (NS) siRNA, and cell numbers were counted on Day 2 and Day 4. **(B, C)** The expression levels of CDH1, KRT14, JUN, CCND1, CCND2, CCN1, and IL6 in CAL 27 cells with or without MORC3 knockdown were analyzed by qRT-PCR. *, *p* < 0.05.

Although MORC3 is an immune-associated gene, RNA-seq analysis indicated the upregulation of several cellular proliferation-related genes, such as JUN, CCND1, CCND2, and cellular communication network factor 1 (CCN1). To evaluate if MORC3 plays a negative role in cancer cell proliferation, cell growth was analyzed in MORC3-deficient cells. MORC3 knockdown significantly promoted the cell growth of CAL 27 cells (Figure 6A). MORC3 deficiency downregulated the E-cadherin (CDH1) tumor suppressive gene and the keratin 14 (KRT14) epithelial differential marker (Figure 6B) but significantly upregulated the expression of oncogenic JUN, CCND1, CCND2, and CCN1 at the transcriptional level (Figure 6C). IL6 is a proinflammatory gene, but it also promotes tumor cell proliferation. MORC3 knockdown increased IL6 expression (Figure 6C). Western blot analysis confirmed that MORC3 knockdown significantly increased the protein expression levels of JUN, CCND1, and CCND2 (Supplementary Figure S7). These results suggested that MORC3 suppresses cancer cell proliferation.

## 4 Discussion

In monocytes, MORC3 binds to a MORC3-regulated element (MRE) near the IFNB1 locus and represses the expression of IFN-β1 (Gaidt et al., 2021). Herpes virus infection triggers the degradation of MORC3 and releases the suppression of IFN-β1 expression, resulting in an anti-viral role (Gaidt et al., 2021). The present study demonstrated that MORC3 repressed the expression of many IFN-associated genes belonging to the IFNα/β/γ signaling pathways. IFNα and IFNβ belong to the type I family of IFNs, which activate IFN-stimulated gene factor 3 complex, including STAT1 and IRF9, and other antiviral target genes. Increased expression of STAT1 and IRF9 enhances the signaling activation (Ivashkiv and Donlin, 2014). The present study showed that MORC3 knockdown significantly increased STAT1 and IRF9 expression, while MORC3 overexpression significantly decreased STAT1 and IRF9 expression, suggesting that MORC3 inhibits type I IFN signaling pathways by repressing STAT1 and IRF9 expression. Moreover, IRF7 expression was significantly increased after MORC3 knockdown. IRF7 is a transcription factor and essential for the expression of type I IFN (Qing and Liu, 2023), and IFN-I in turn promotes IRF7 expression, forming a positive feedback loop. These results suggested that MORC3 inhibits IFNβ expression via suppressing IRF7 expression and interrupting the positive feedback loop. However, the present RNA-seq results did not confirm the increased mRNA levels of type I IFNs, such as IFNα and IFNβ, after MORC3 knockdown and IRF7 upregulation in OSCC cells, suggesting that other factors tightly control the expression of type I IFNs.

Type I IFN has a dual role in cancers. Strong IFN responses can mediate anticancer effects, whereas weak and persistent IFN responses can support tumorigenesis (Holicek et al., 2024). IRF7 can play either tumor suppressive or oncogenic roles in different cancers (Qing and Liu, 2023). The tumor suppressive function of IRF7 is mainly related to the production of IFNβ, but IRF7 also promotes the M2 polarization of tumor-associated macrophages and enhances tumor immune evasion and proliferation (Tu et al., 2021). In head and neck cancer, both IRF7 and IRF9 have been reported to be overexpressed (Liu and Wang, 2022). IFIT1 and IFITM3 are highly expressed and associated with poor prognosis in head and neck cancer (Li et al., 2020). IFIT1 promotes the metastasis in OSCC (Pidugu et al., 2019). The present study demonstrated that IRF7 expression was negatively correlated with MORC3 in single head and neck cancer cells, which was correlated with the MORC3-mediated suppression of IRF7 expression. Thus, MORC3 may play important roles in suppressing the persistent activation of IFN-associated pathways, which may block the initiation and development of cancer.

MORC2, a MORC family member, is considered as an oncogenic protein because it is highly expressed in cancers and promotes the migration, invasion, and metastasis of cancer cells (Wang et al., 2021). However, MORC3 activates the p53 tumor suppressor and induces cellular senescence (Takahashi et al., 2007). In cancers, the function of MORC3 may be disturbed by an R420Q mutation in the CW domain, which decreases the binding of MORC3 to H3K4me3 (Andrews et al., 2016). In addition, dermatomyositis patients with anti-MORC3 autoantibody have an increased frequency of cancer compared to those without the anti-MORC3 autoantibody (Andrews et al., 2016). The present study demonstrated that DDX60 expression was suppressed by MORC3. A recent study has demonstrated that DDX60 is upregulated in pancreatic cancer, and DDX60 promotes cancer cell proliferation and is associated with poor patient survival (Lai et al., 2023). MORC3 also decreases the expression of OAS1, which is overexpressed in multiple cancers (Jiang et al., 2023). The present study demonstrated that MORC3 decreases PD-L1 expression. In summary, these findings suggested that MORC3 may act as tumor suppressor.

Single-cell RNA-seq technology is a novel method used to profile the whole transcriptome at the single-cell level (Conte et al., 2024). The present study used the online CancerSEA database to analyze the expression of MORC3 and its target genes in head and neck cancer cells, which demonstrated a negative correlation between MORC3 and IFN-associated target genes, as well as between MORC3 and PD-L1. The single-cell RNA-seq results were confirmed by qRT-PCR and bulk RNA-seq. These findings suggested that MORC3 is expressed with its target genes within the same cancer cell.

PD-L1 is a transmembrane protein expressed on the surface of dendritic cells and lymphocytes. Tumor cells in many types of cancer, including head and neck cancer, also express PD-L1. By interacting with PD-1 on the surface of T cells, tumor cells inhibit the activation of T cells and evade immune attack (Lin et al., 2023). Multiple pathways have been reported to control PD-L1 expression at the transcriptional level. For example, DNA double-stand breaks increase PD-L1 expression by activating the STAT1/STAT3 signaling pathway (Sato et al., 2017), and the phosphatidylinositol 3-kinase (PI3K) pathway activates PD-L1 expression through AKT and is negatively regulated by phosphatase and tensin homolog (PTEN) (Song et al., 2013). The present study demonstrated that MORC3 is a novel suppressor of PD-L1 expression. MORC3 knockdown increased STAT1 expression, whereas MORC3 overexpression reduced STAT1 expression. Thus, MORC3 may inhibit PD-L1 expression by suppressing STAT1 expression. Moreover, MORC3 inhibited the expression of the oncogenic LINC00880 lncRNA. LINC00880 knockdown represses cancer cell proliferation, colony formation, migration, and *in vivo* tumor formation. Mechanically, LINC00880 interacts with the cyclin-dependent kinase 1 (CDK1) and peroxiredoxin-1 (PRDX1) proteins to enhance the kinase activity of CDK1, the interaction between CDK1 and PRDX1, and the phosphorylation of PRDX1, thereby leading to the activation of the PI3K/AKT signaling pathway (Feng et al., 2023). The present results suggested that MORC3 is an upstream negative regulator of LINC00880 expression, and LINC00880 may promote immune evasion by promoting PD-L1 expression. More experiments are required to understand the molecular mechanisms of the MORC3/LINC00880/PD-L1 regulatory axis.

In summary, MORC3 regulates IFN-associated pathways and is a novel repressor of PD-L1 expression through multiple pathways.

## Data Availability

The RNA-seq data have been deposited in the GEO database (GSE254613).
