# Supplementary material for: Genome-wide analysis reveals the MORC3-mediated repression of PD-L1 expression in head and neck cancer
Source: Front Cell Dev Biol. 2024 Sep 12;12:1410130. doi: 10.3389/fcell.2024.1410130 (PMC11425343; doi:10.3389/fcell.2024.1410130)
Supplement: Supplementary file 1 [file Table1.DOCX]

**Table S1.** The clinical characteristics of OSCC patients and normal controls in tissue microarray.

a. OSCC

| Characteristics | Number of Cases (%) |
| --- | --- |
| Age (Y) |  |
| <55 | 22 (45.8%) |
| ≥55 | 26 (54.2%) |
| Gender |  |
| Male | 28 (58.3%) |
| Female | 20 (41.7%) |
| Stage |  |
| I | 9 (18.75%) |
| I + II | 19 (39.6%) |
| II | 13 (27.1%) |
| III and II+ III | 4 (8.3%) |
| Unknown stage | 3 (6.25%) |

b. Normal

| Characteristics | Number of Cases (%) |
| --- | --- |
| Age (Y) |  |
| <55 | 4 (40%) |
| ≥55 | 6 (60%) |
| Gender |  |
| Male | 6 (60%) |
| Female | 4 (40%) |

**Table S2.** Primers for qPCR.

| **Gene** | **Primers** |
| --- | --- |
| **DDX60** | 5’-CTGGTTGAACGCTATCTTGTGG-3’  5’-GAACATCAATGGTGGTATTCTTCTG-3’ |
| **IFI44L** | 5’-TATCACCAGCATAACCGAGCG-3’  5’-GTCATCCATGCACAGTCCTGC-3’ |
| **IFIT2** | 5’-CCTGGAACTTGATGGAGGGAG-3’  5’-GCCCTTTGAGGTGCTTTAGATAG-3’ |
| **USP18** | 5’-CATCCTGGCTGAGTCCTCGC-3’  5’-CAGCAGGTCTGTCCAATGTTGTG-3’ |
| **MX2** | 5’-GTCTCGCCAACCAGATCCCA-3’  5’-CGGTCTCACTCTGCTCTTGAAGC-3’ |
| **IFIT1** | 5’-TCAGGTCAAGGATAGTCTGGAGC-3’  5’-CAGGTGTTTCACATAGGCTAGTAGG-3’ |
| **IRF7** | 5’-CCCACGCTATACCATCTACCT-3’  5’-GATGTCGTCATAGAGGCTGTTG-3’ |
| **IRF9** | 5’-GCCCTACAAGGTGTATCAGTTG-3’  5’-TGCTGTCGCTTTGATGGTACT-3’ |
| **IFITM3** | 5’-CGCTGGTCTTCGCTGGACAC-3’  5’-CACAGCCACCTCGTGCTCCT-3’ |
| **IFITM1** | 5’-CCAAGGTCCACCGTGATTAAC-3’  5’-ACCAGTTCAAGAAGAGGGTGTT-3’ |
| **IFI44** | 5’-TTTTCGATGCGAAGATTCACTGG-3’  5’-CCTGATGCGTTACATGCCCTT-3’ |
| **IFIH1** | 5’-TCACAAGTTGATGGTCCTCAAGT-3’  5’-CTGATGAGTTATTCTCCATGCCC-3’ |
| **STAT1** | 5’-CATGGAAATCAGACAGTACCTGGC-3’  5’-AGACATCTGGATTGGGTCTTCCTG -3’ |
| **PD-L1** | 5’-ATGCCTTGGTGTAGCACTGA-3’  5’-GCTGGATTACGTCTCCTCCAAA-3’ |
| **CDH1** | 5’-TGCCCAGAAAATGAAAAAGG-3’  5’-GTGTATGTGGCAATGCGTTC -3’ |
| **KRT14** | 5’-GGAGATGATTGGCAGCGTGGAG-3’  5’-AGAACTGGGAGGAGGAGAGGTG-3’ |
| **JUN** | 5’-CAAGAACTCGGACCTCCTCA-3’  5’-TCCTGCTCATCTGTCACGTT-3’ |
| **CCND1** | 5’-CAATGACCCCGCACGATTTC-3’  5’-CATGGAGGGCGGATTGGAA-3’ |
| **CCND2** | 5’-GAGAAGCTGTCTCTGATCCGCA-3’  5’-CTTCCAGTTGCGATCATCGACG-3’ |
| **CCN1** | 5’-CGCCTTGTGAAAGAAACCCG-3’  5’-GGTTCGGGGGATTTCTTGGT-3’ |
| **IL6** | 5’-ACTCACCTCTTCAGAACGAATTG-3’  5’-CCATCTTTGGAAGGTTCAGGTTG-3’ |
