# Supplementary material for: Genome-wide analysis reveals the MORC3-mediated repression of PD-L1 expression in head and neck cancer
Source: Front Cell Dev Biol. 2024 Sep 12;12:1410130. doi: 10.3389/fcell.2024.1410130 (PMC11425343; doi:10.3389/fcell.2024.1410130)
Supplement: Supplementary file 2 [file Image1.PDF]

Supplementary Figure S1

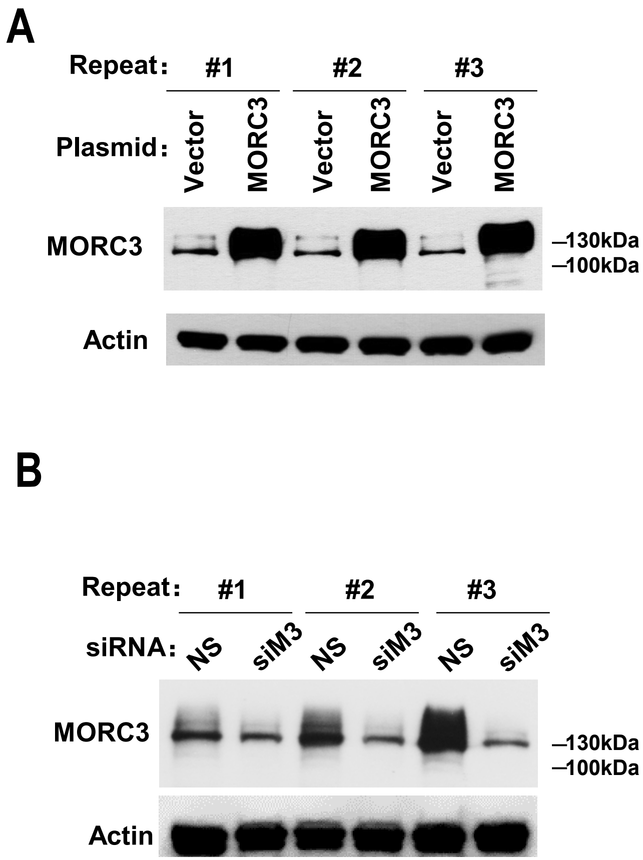

Supplementary Figure S1. The overexpression (A) and knockdown (B) efficiency of MORC3 were confirmed by western blot in CAL 27 cells.

Supplementary Figure S2

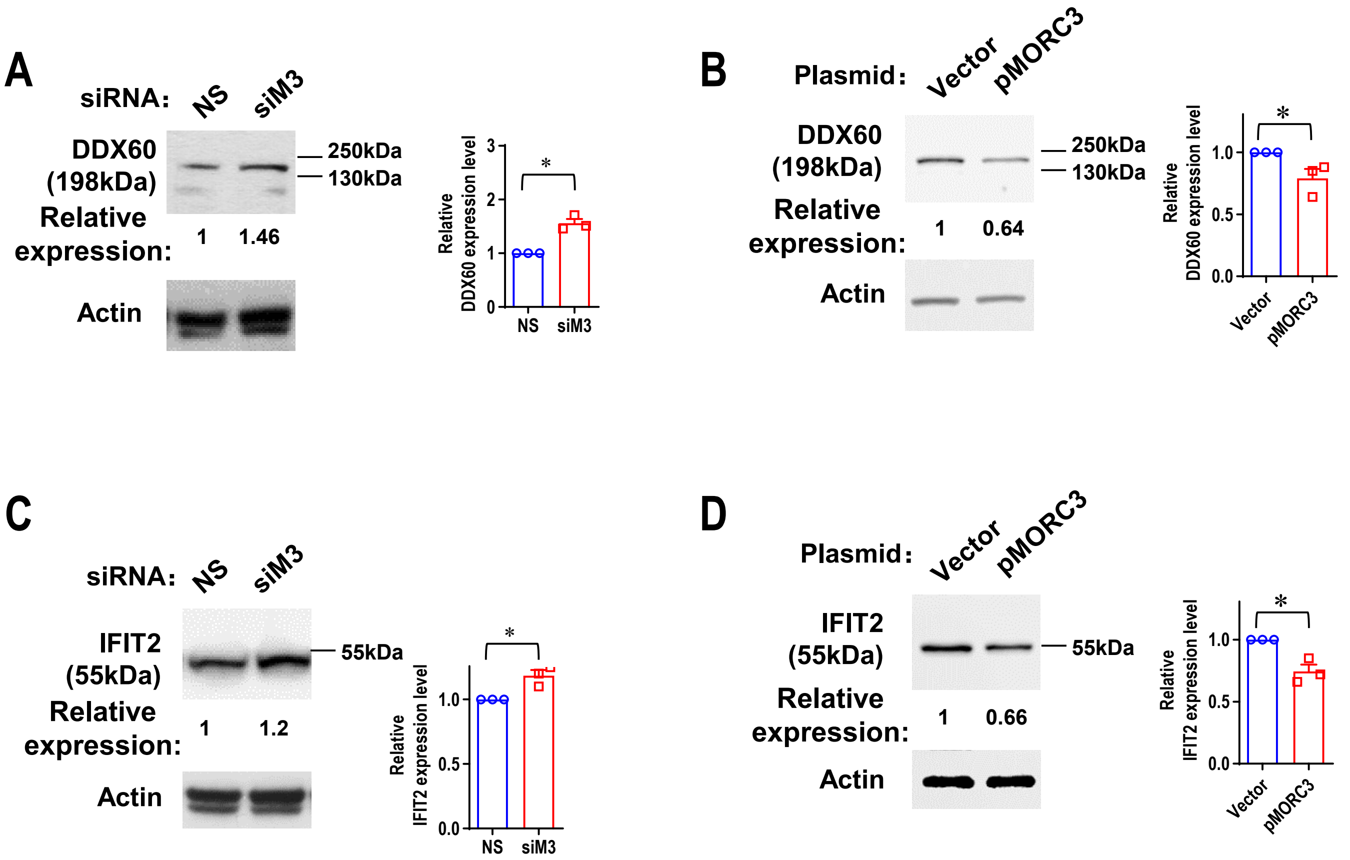

Supplementary Figure S2. The effects of knockdown (A, C) and overexpression (B, D) of MORC3 on the expression DDX60 and IFIT2 were confirmed by western blot in CAL 27 cells. Actin serves as a loading control. The histograms on the right summarized the quantification results of western blot (n=3).

Supplementary Figure S3

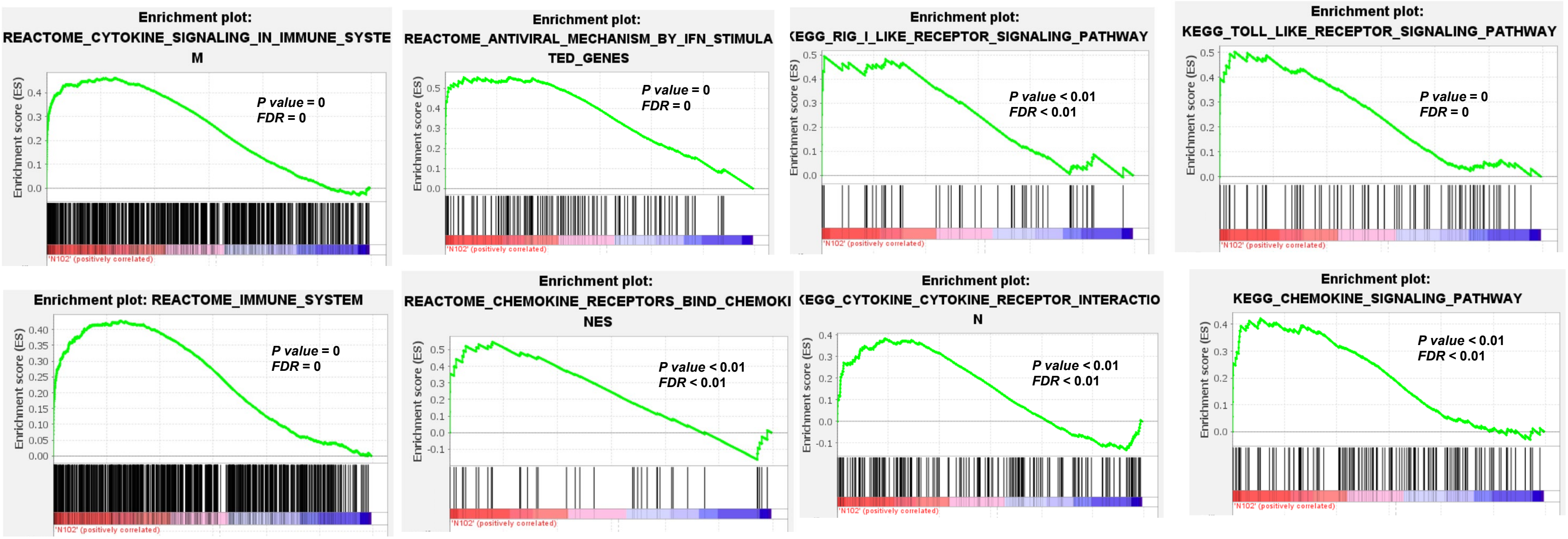

Supplementary Figure S3. The GSEA analysis of other immune-related signaling pathways enriched in Reactome and KEGG analyses.

Supplementary Figure S4

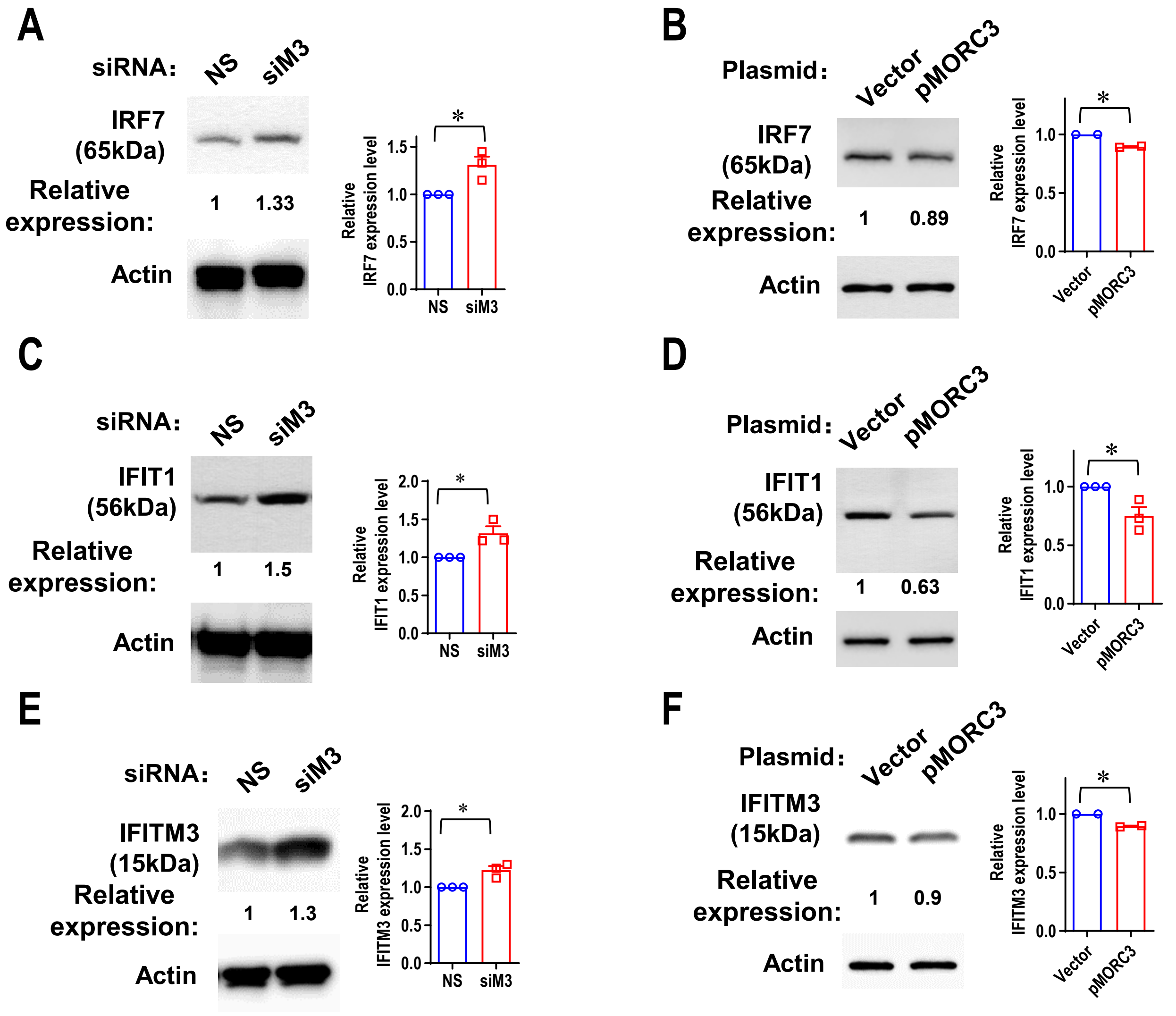

Supplementary Figure S4. The effects of knockdown (A, C, E) and overexpression (B, D, F) of MORC3 on the expression IRF7, IFIT1, and IFITM3 were confirmed by western blot in CAL 27 cells. Actin serves as a loading control. The histograms on the right summarized the quantification results of western blot (n=2 or 3).

Supplementary Figure S5

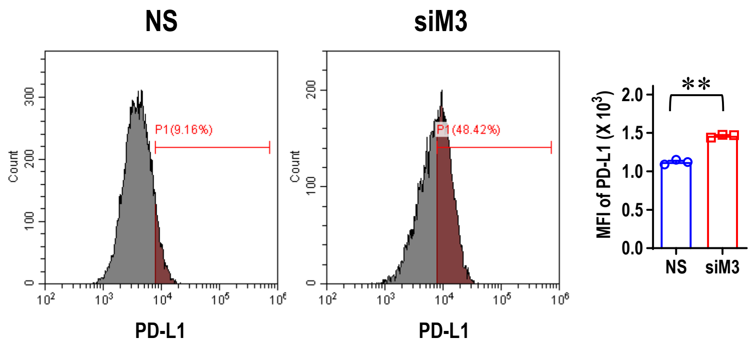

Supplementary Figure S5. The expression of PD-L1 in CAL 27 cells treated with anti-MORC3 (siM3) or non-specific (NS) siRNA was analyzed by flow cytometry. The histograms on the right summarized the quantification results of western blot (n=3).

Supplementary Figure S6

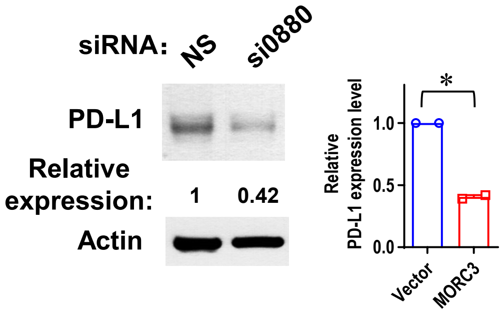

Supplementary Figure S6. The effect of LINC00880 knockdown on the expression PD-L1 was confirmed by western blot in CAL 27 cells. Actin serves as a loading control. The histogram on the right summarized the quantification results of western blot (n=2).

Supplementary Figure S7

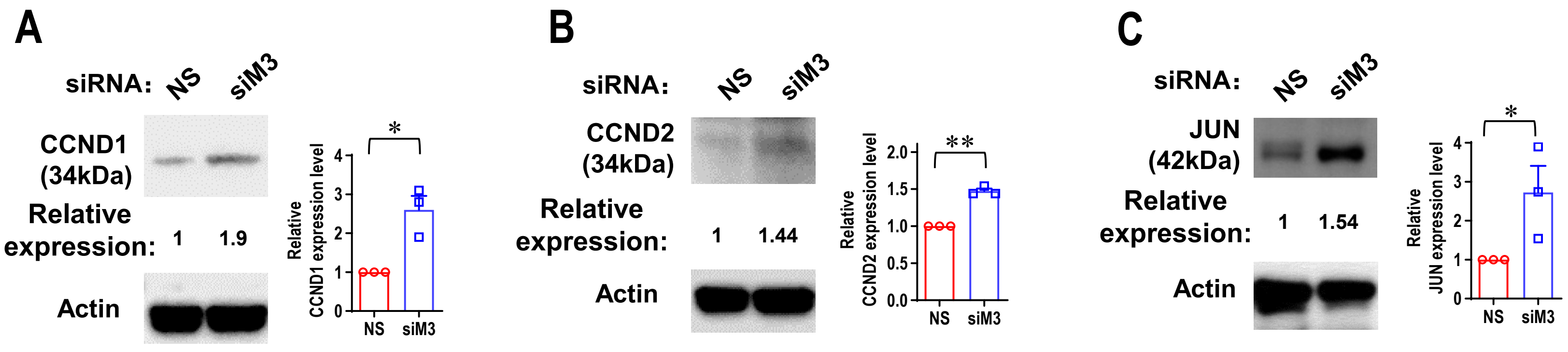

Supplementary Figure S7. The effects of MORC3 knockdown on the expression CCND1, CCND2, and JUN were confirmed by western blot in CAL 27 cells. Actin serves as a loading control. The histograms on the right summarized the quantification results of western blot (n=3).
